# Long Non-coding RNA MALAT1 Is Depleted With Age in Skeletal Muscle *in vivo* and MALAT1 Silencing Increases Expression of TGF-β1 *in vitro*

**DOI:** 10.3389/fphys.2021.742004

**Published:** 2022-01-21

**Authors:** Ling Ruan, Bharati Mendhe, Emily Parker, Andrew Kent, Carlos M. Isales, William D. Hill, Meghan McGee-Lawrence, Sadanand Fulzele, Mark W. Hamrick

**Affiliations:** ^1^Medical College of Georgia, Augusta University, Augusta, GA, United States; ^2^Department of Pathology and Laboratory Medicine, Medical University of South Carolina, Charleston, SC, United States

**Keywords:** sarcopenia, siRNA, senescence, oxidative stress, fibrosis

## Abstract

Long non-coding RNAs (lncRNAs) are thought to function as “sponges” for microRNAs, but a role for such competing endogenous RNAs (ceRNAs) in muscle aging is not well understood. We therefore examined in skeletal muscles of young (4–6 months) and aged (22–24) male and female mice the expression of lncRNA MALAT1, which is predicted *in silico* to bind the senescence-associated microRNA miR-34a-5p. Results indicate a significant decrease in lncRNA MALAT1 expression in mouse skeletal muscle with age that coincides with an age-related increase in miR-34a-5p expression. *In vitro* studies using mouse C2C12 myoblasts demonstrate that MALAT1 silencing using siRNA increases miR-34a expression, consistent with a role for MALAT1 as an inhibitor of miR-34a-5p activity. Levels of reactive oxygen species (ROS) are known to increase in muscle with age, and so we treated C2C12 cells with hydrogen peroxide (10 and 100 μM) to examine changes in MALAT1 expression. MALAT1 expression decreased significantly with H_2_O_2_ treatment, but this effect was attenuated with p53 siRNA. Finally, miR-34a-5p is implicated in tissue fibrosis, and so we assessed the expression of TGF-β1 after MALAT1 silencing. MALAT1 siRNA significantly increased the expression of TGF-β1 in C2C12 cells. These findings suggest that age-related fibrosis and muscle atrophy mediated by ROS may result at least in part from an increase in miR-34a bioavailability resulting from a decline in miR-34a “sponging” due to ceRNA MALAT1 depletion. Crosstalk between MALAT1 and miR-34a may therefore represent a therapeutic target for improving muscle function with aging.

## Introduction

The loss of muscle mass with age, or sarcopenia, is a significant clinical concern as declines in muscle mass are associated with frailty and disability among older adults. The mechanisms underlying the development and progression of sarcopenia are multifactorial but are becoming better understood. Increased inflammation with aging can increase the activity of ubiquitin ligases such as atrogin-1 that catabolize muscle proteins (Livshits and Kalinkovich, 2019), and increased oxidative stress with aging is associated with cellular senescence, mitochondrial dysfunction, and ultimately muscle loss (Thoma et al., 2020). Previous research suggests that lncRNAs may play important roles in muscle growth and muscle cell differentiation. The recently identified long non-coding RNA Chronos was found to be upregulated with age, and Chronos inhibition can induce myofiber hypertrophy both *in vitro* and *in vivo* (Neppl et al., 2017). Additional lncRNAs such as Neat1, Malat1, Sra, Meg3, and LncMyoD show distinct patterns of expression during myoblast differentiation, suggesting key roles for these non-coding RNAs in muscle fiber development and maturation (Butchart et al., 2016).

Long non-coding RNAs (lncRNAs) are known to function as “sponges” for microRNAs (Paraskevopoulou and Hatzigeorgiou, 2016), but a role for such competing endogenous RNAs (ceRNAs) in muscle aging is not well understood. We have recently shown that increased levels of reactive oxygen species (ROS) in aging skeletal muscle are associated with increased expression of the senescence-associated microRNA miR-34a-5p (miR-34a) (Fulzele et al., 2019). The histone deacetylase Sirt1 is a validated target of miR-34a, and miR-34a expression is induced by the tumor suppressor p53 which is itself stimulated by ROS. We therefore examined in skeletal muscles of young (4–6 months) and aged (22–24) male and female mice the expression of several lncRNAs that are predicted to bind miR-34a-5p *in silico* and whose predicted binding has been validated experimentally. We also examined the impact of oxidative stress on lncRNA expression. Our results indicate that MALAT1 can modulate miR-34a *in vitro* and that MALAT1 is depleted with age and oxidative stress. These findings suggest that therapeutic strategies increasing MALAT1 expression in muscle with aging may improve muscle health with aging.

## Materials and Methods

### *In silico* Prediction of Competing Endogenous RNA Binding of mmu-miR-34a-5p

**FIGURE 1:**
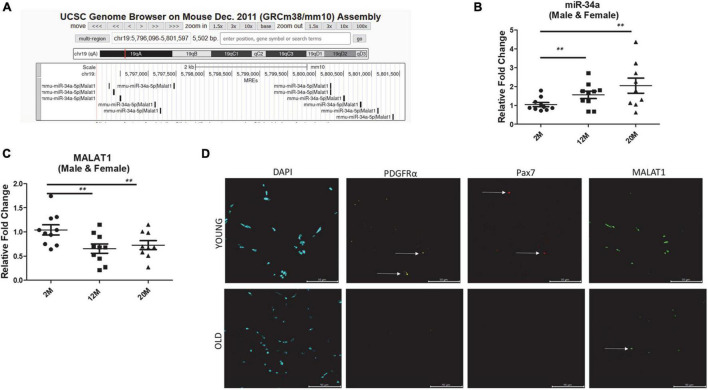
**(A)** microRNA response elements (MREs) shown for mouse chromosome 19 region of Malat1 illustrating numerous binding sites for miR-34a-5p. **(B)** Expression of miR-34a-5p in tibialis anterior muscles of male and female mice 2, 12, and 24 months of age showing a significant increase in mir-34a-5p expression with age. **(C)** Expression of MALAT1 in tibialis anterior muscles of male and female mice 2, 12, and 24 months of age showing a significant decrease in MALAT1 expression with age. **(D)** Fluorescent images from tibialis anterior cross-sections stained using RNAscope with probes for MALAT1, PDGFRα, and Pax7 showing decreased MALAT1 levels with age. Arrows in **(D)** indicate positive labeling. **P < 0.01.

Our initial goal was to identify lncRNAs that might “sponge” miR-34a-5p and thus alter its capacity to bind downstream targets such as Sirt1. We used LncBase Experimental v.2 module in DIANA tools (Paraskevopoulou et al., 2016) to identify lncRNAs that were verified experimentally as binding miR-34a. The lncRNAs identified in this initial screen included Gas5, Hotair, Malat1, Snhg6, Tug1, and Tbrg3 with prediction scores ranging from 0.38 to 0.70, respectively (Table 1). We then used PubMed to further refine this list to include those candidates that were previously identified in skeletal muscle. Malat1 retrieved the greatest number of hits (21), whereas all others only retrieved 0–2 hits (Table 1). In a search using the lncRNA terms + aging Malat1 also received the largest number of hits (Table 1). We then queried the UCSC genome browser (Kent et al., 2002) for microRNA response elements (MREs) for miR-34a-5p in the mouse MALAT1 sequence. Results demonstrate a number of predicted binding sites for miR-34a-5p in mouse MALAT1 (Figure 1A). All subsequent *in vivo* and *in vitro* experiments therefore focused on MALAT1.

**TABLE 1:** DIANA prediction scores for mmu-miR-34a-5p lncRNA binding and PubMed searches for various search terms and lncRNAs.

lncRNA	Prediction Score	lncRNA + skeletal muscle	lncRNA + aging
Snhg6	0.38	0	0
Hotair	0.39	2	15
Gas5	0.39	0	14
Malat1	0.53	21	37
Tug1	0.62	2	7
Tbrg3	0.70	0	0

### MALAT1 and mir-34a-5p Expression in Aged Mice

Five male and five female C57BL6 mice were obtained from the National Institute on Aging at 2, 12, and 20 months of age. Mice were euthanized by CO_2_ overdose and thoracotomy following IACUC approved procedures. The tibialis anterior muscle was dissected free and snap frozen in liquid nitrogen prior to isolation of miRNA and lncRNA. Total RNA was isolated by TRIzol method and miRNA isolation using the miRNeasy kit (Qiagen) following manufacturer specifications. RNA was reverse transcribed into using iScript reagents from Bio-Rad on a programmable thermal cycler. 50 ng of cDNA was amplified in each real-time PCR using a Bio-Rad iCycler using the following primer sequences: GAPDH Rev 5′-CTCGCTCCTGGAAGATGGTG-3′, GAPDH Fwd 5′ GGTGAAGGTCGGTGTGAACG-3′, 18S Rev 5′-GGGCCTCACTAAACCATCCA-3′, 18S Fwd 5′-AGTGCCGGTCATAAGCTTGC-3′, TGF-β1 Rev 5′-GCC ACT GCC CAT CGT CTA CT-3′, TGF-β1 Fwd 5′-CAC TTG CAG GAG CGC ACA AT-3′, MALAT1 Rev 5′-AAC TAC CAG CAA TTC CGC CA-3′, MALAT1 Fwd 5′-GAG CTC GCC AGG TTT ACA GT-3′. The MALAT1 primer (Accession number FJ209304) anneals at bases 2168–2264, and the product size is 96 bp. This sequence amplifies the 3′ end of the Malat gene (upper 33% of the 3′ region). The average expression of housekeeping genes glyceraldehyde-3-phosphate dehydrogenase (GAPDH) and 18S RNA were used as the internal controls for normalization. For miRNA-34a quantitation, miRNA was isolated using the miRNA easy Isolation kit and reverse-transcribed into cDNA using miScript reagents (Qiagen). 50 pg of cDNA was amplified in each qRT-PCR using SYBR Green I and miR-34a primers (Qiagen, MS00001428). The average of RNU6 (Qiagen, MS00033740) and SNORD (MS00033705) was used to normalize miR-34a expression.

### RNAscope Localization of MALAT1 Expression *in situ*

We confirmed the PCR data on MALAT1 from homogenized muscle using *in situ* labeling of MALAT1 with RNAscope (ACD Bio) technology. Slides from two males and two females per age group (2 months and 20 months) were used for staining. The MALAT1 probe (Accession No. NR_002847.2, Target Region: 712–2338) is labeled with Opal Dye 520, Pax7 (NM_011039.2, Target Region: 602–1534) labeled with Opal Dye 690, and PDGFRα (NM_011058.2, Target Region: 223–1161) labeled with Opal Dye 620. Slides were submerged in xylene and 100% EtOH then treated with hydrogen peroxide for 10 min at room temperature. The slides were then placed in pretreatment (ACD Bio) buffer and boiled at 99°C for 30 min using a rice cooker. The slides were incubated with protease plus (ACD Bio) for 30 min at 40°C in the EZ Hybrid Oven (ACD Bio). RNAscope multiplex assay protocol was performed according to manufacturer instructions. In brief, the probe mixtures (ACD Bio) were placed on the slides for a 2-h incubation at 40°C. Afterward, the slides were incubated overnight at 4°C in 5X SSC buffer (Millipore Sigma). The slides were then incubated with AMP1, AMP2 and AMP3 (ACD Bio) at 40°C. This was followed by incubation of HRP-C1 (ACD Bio), followed by the corresponding opal dye, followed by HRP block (ACD Bio). Opal dye 570 (Akoya Biosciences) was used and diluted in TSA dilution buffer (ACD Biosciences). Once complete, mounting media with DAPI (Vector Labs) was added and the slide covered with a cover slip. Slides were imaged using confocal microscopy.

### MALAT1 and p53 siRNA

Experiments utilizing siRNA to p53 and MALAT1 were performed in C2C12 cells seeded on 6-well plates. Samples were in triplicate for transfection studies. Lipofectamine RNAiMAX Transfection Reagent (Cat. #13-778-150, Invitrogen) was used for transfection. Silencer Select Negative Control No. 1 siRNA (Cat. #4390843, Ambion) was used as a control siRNA and Silencer Select MALAT1 siRNA, (siRNA ID # n253517, Ambion) used for MALAT1 transfection. siRNA for P53 was from Santa Cruz (Cat. #SC-29436). In brief, cells were monitored after 48 h in culture and transfection performed on 75–80% confluent cells for optimal results. Media was replaced and 750 μl of Opti-MEM added to each well before transfection. 3 μl of 10 μM Control, p53, or MALAT1 siRNA was used for transfection. Cells were harvested 48 or 72 h after transfection and H_2_O_2_ (10 μM or 100 μM) treatment to perform analysis.

### Cell Imaging and Myotube Differentiation

Transfected C2C12 cells were seeded and cultured in DMEM with 10% FBS. Once cells reached 80% confluence, media was changed to DMEM with 2% horse serum to induce differentiation. Cells were cultured in the presence of horse serum for 96 h before staining and imaging. Cells were fixed with 4% paraformaldehyde (PFA) for 7 min, washed with 1X PBS for three times, blocked in blocking buffer (2% bovine serum albumin, 0.2% normal goat serum, and 0.05% Triton-X 100) for 30 min, then incubated for 3 h or overnight with Alexa Fluor™ 488 Myosin 4 antibody. Cells were washed three times with 1X PBS, slides mounted with DAPI mounting medium, and sealed with a coverslip. Cells were imaged using confocal microscopy (Leica STELLARIS) and the fusion index calculated as the number of nuclei within myotubes/total number of visible nuclei for approximately five images per treatment group.

### Statistical Analysis

GraphPad Prism 9.2 (La Jolla, CA, United States) was utilized to perform ANOVA with Bonferroni pair-wise comparison or unpaired *t*-tests as appropriate. *P*-values of <0.05 were considered significant.

## Results

*In silico* analysis of microRNA response elements (MREs) shown for mouse chromosome 19 region of Malat1 demonstrate numerous binding sites for miR-34a-5p (Figure 1A). Analysis of miR-34a-5p gene expression in skeletal muscle from male and female young and aged mice shows that miR-34a expression increases with age in skeletal muscle (Figure 1B). On the other hand, analysis of the same samples indicates that MALAT1 expression decreases significantly with age (Figure 1C). Fluorescent images from tibialis anterior cross-sections stained using RNAscope with probes for MALAT1 confirm the PCR results and indicated decreased MALAT1 levels with age (Figure 1D). Few MALAT1 positive cells co-localize with either PDGFRα, a marker of fibro-adipogenic progenitor cells, or with Pax7, a marker of muscle satellite cells, suggesting that MALAT1 is expressed primarily by myonuclei (Figure 1D).

**FIGURE 2:**
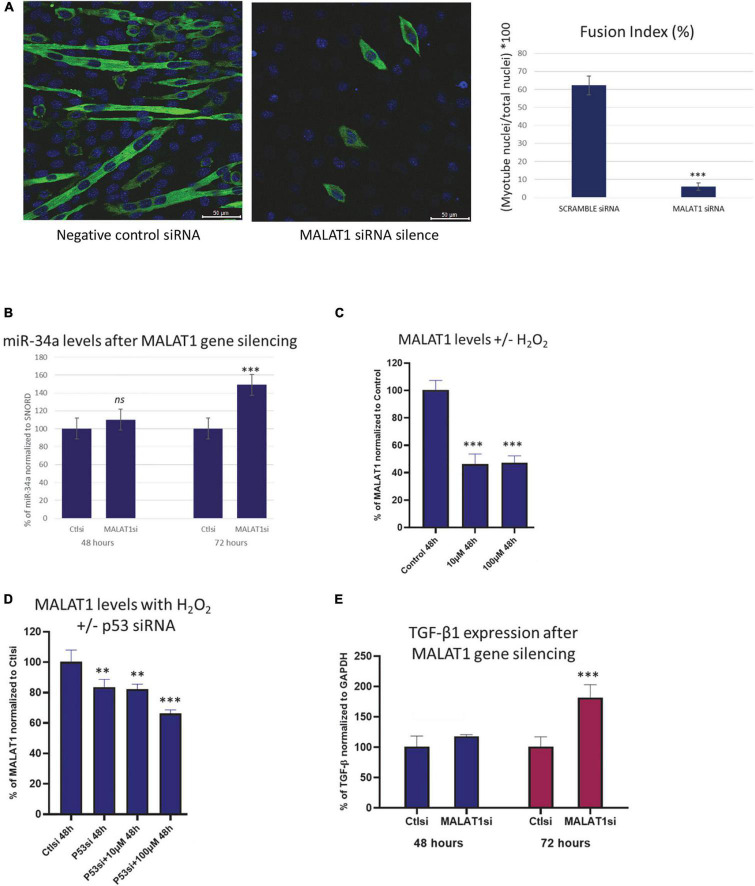
**(A)** Confocal images of C2C12 cells cultured in differentiation medium transfected with scramble (control) siRNA (left) or MALAT1 siRNA (right) showing impaired differentiation with MALAT1 silencing. **(B)** Expression levels of miR-34a-5p in C2C12 mouse myoblasts transfected with scramble control or with MALAT1 siRNA (MALATsi). siRNA transfection is effective after 72 h, and shows increased miR-34a-5p levels with MALAT1 inhibition. **(C)** Decreased expression of MALAT1 in C2C12 mouse myoblasts after exposure to hydrogen peroxide (H_2_O_2_). **(D)** The decrease in MALAT1 expression after hydrogen peroxide exposure is attenuated with p53 inhibition using siRNA (p53si). **(E)** Expression levels of TGF-β1 in C2C12 mouse myoblasts transfected with scramble control or with MALAT1 siRNA (MALATsi). siRNA transfection is effective after 72 h, and shows increased TGF-β1 levels with MALAT1 inhibition. *ns P* > 0.05, ^**^P < 0.01, ^***^P < 0.001. All assays were performed in triplicate.

*In vitro* experiments utilizing mouse C2C12 myoblasts transfected with MALAT1 siRNA show that normal myotube maturation and differentiation is impaired with MALAT1 silencing (Figure 2A). The inhibition of differentiation is reflected in the significantly lower fusion index following MALAT1 silencing (Figure 2A). Expression levels of miR-34a-5p in these cells are increased with MALAT1 inhibition (Figure 2B). Reactive oxygen species can increase mir-34a expression, and so we treated C2C12 cells with hydrogen peroxide (H_2_O_2_) to examine effects of oxidative stress on MALAT1. These experiments show that H_2_O_2_ treatment significantly decreases MALAT1 expression by approximately 60% at both low (10 μM) and high (100 μM) doses (Figure 2C). We then repeated these experiments in cells transfected with p53 siRNA. These experiments show that p53 silencing attenuates the effects of H_2_O_2_ treatment on MALAT1 expression, such that the decrease in expression is ∼20% as opposed to the 60% decline observed without p53 silencing (Figure 2D). Cellular senescence and oxidative stress are associated with fibrosis in a number of different organs and tissues, and so we examined effects of MALAT1 silencing on TGF-β1 and expression. Results show that MALAT1 silencing significantly increases the expression of TGF-β1 in mouse C2C12 myoblasts (Figure 2E).

## Discussion and Conclusion

A role for MALAT1 in skeletal muscle growth and development was first indicated by the finding that treatment of mice with recombinant myostatin significantly downregulated MALAT1 expression (Watts et al., 2013). It was later found that MALAT1 is highly expressed in the later stages of muscle cell differentiation, during myoblast fusion and myotube hypertrophy (Butchart et al., 2016; Chen et al., 2017). The expression of MALAT1 in muscle cells tends to parallel the expression of myogenin, and MALAT1 silencing decreases myogenin expression (Watts et al., 2013). These previous studies are consistent with our results demonstrating that MALAT1 silencing impairs normal myotube maturation (Figure 2A). Consistent with the finding that MALAT1 is suppressed with myostatin treatment, MALAT1 expression in skeletal muscle is decreased with hindlimb unloading and glucocorticoid treatment (Hitachi et al., 2020). On the other, recent data also show that exercise increases MALAT1 expression in muscles of older adults (De Sanctis et al., 2021). These data would suggest that MALAT1 depletion is associated with conditions associated with muscle atrophy and increased in settings of muscle anabolism. Indeed, although Butchart et al. (2016) observed that MALAT1 lncRNAs remained stable during post-natal growth, Mikovic et al. (2018) did identify a decrease in MALAT1 between 4 weeks and 28 months of age, consistent with our data shown in Figure 1.

**FIGURE 3:**
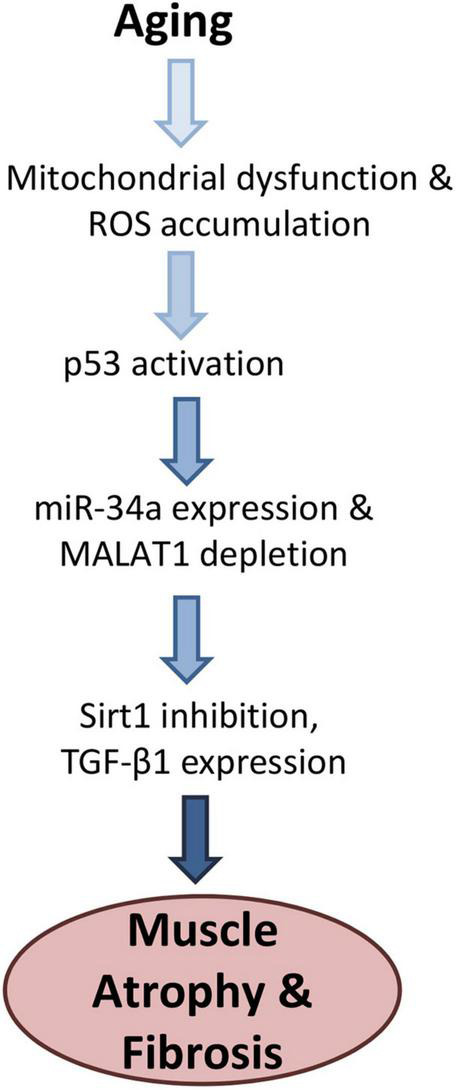
Proposed model for age-related changes linking oxidative stress with increased miR-34a-5p expression and Malat1 inhibition. Activation of p53 both stimulates miR-34a and suppresses MALAT1, which creates a feed-forward cycle further increasing miR-34a levels. Elevated mir-34a targets Sirt1 which can increase senescence as well as TGF-β1, increases fibrosis. Together these factors are likely to impair muscle function with aging.

We previously found that the senescence-associated microRNA mir-34a-5p is increased with aging in mouse skeletal muscle as well as in muscle-derived exosomes (Fulzele et al., 2019). Here we observed an inverse relationship between miR-34a and MALAT1 with aging, a similar relationship that has previously been observed in melanoma cells and in melanoma tumor tissue (Li et al., 2019). We also found that miR-34a can be suppressed by MALAT1, which has also been documented by luciferase assay in cancer cells (Li et al., 2019) and other cell types (Duan et al., 2019; Sun et al., 2019). These findings are significant as they suggest that crosstalk between miR-34a and MALAT1 may represent a therapeutic target for improving muscle function with aging. Mir-34a is elevated with aging in tissues ranging from cardiac muscle (Boon et al., 2013) to skeletal muscle (Zheng et al., 2018; Fulzele et al., 2019), articular cartilage (Jeon et al., 2019) and circulating blood cells (Owczarz et al., 2017). Sirt1 is a well-established target of miR-34a, Sirt1 declines in muscle with age (Myers et al., 2019), and suppression of Sirt1 by miR-34a can induce senescence (Badi et al., 2015). Importantly miR-34a is also secreted from senescent cells via exosomes (Fulzele et al., 2019; Jeon et al., 2019), and so it is an important component of the senescence-associated secretory phenotype (SASP). Our finding that ROS-induced oxidative stress decreased MALAT1 is consistent with a role for MALAT1 as an inhibitor of miR-34a activity. In addition, MALAT1 silencing in fibroblasts can itself induce senescence and increase expression of p16 and p21 (Tripathi et al., 2013). Mir-34a is induced by ROS through p53 activation (Raver-Shapira et al., 2007), p53 is increased in skeletal muscle with aging (Edwards et al., 2007), and p53 also acts as a transcriptional repressor of MALAT1 (Ma et al., 2015). We found that ROS exposure (hydrogen peroxide) suppressed MALAT1 expression by ∼60%, but this effect was attenuated with p53 siRNA. These data suggest that interactions and crosstalk between mir-34a and MALAT1 are mediated in large part by p53 activity (Figure 3).

Senescence is known to induce tissue fibrosis, and miR-34a can stimulate fibrosis in a variety of cell and tissue types whereas miR-34a inhibition can inhibit fibrosis (Cui et al., 2017; Wan et al., 2017; Liu et al., 2019). Fibrosis increases with age in skeletal muscle (Lin et al., 2018), and increased age is associated with muscle fiber atrophy (Kaiser et al., 2019) and increased expression of TGF-β1 (Carlson et al., 2008; Parker et al., 2017). TGF-β1 can not only increase fibrosis in skeletal muscle, but it is a key factor that impairs the regenerative capacity of satellite cells with aging (Carlson et al., 2008). Muscles of aged mice also show increased TGF-β1 expression near the neuromuscular junction, suggesting that TGF- β1 could also play a role in the neuromuscular junction degradation and fragmentation that occurs with aging (Blasco et al., 2020). Our finding that MALAT1 silencing increased expression TGF-β1 is likely explained by the increase in miR-34a expression that we also observed with MALAT1 inhibition. Previous studies linking miR-34a to increased fibrosis have explained this association within the context of Sirt1 inhibition by miR-34a (Cui et al., 2017), although inhibition of Klotho may also play a role (Liu et al., 2019). Sirt1 and/or Klotho inhibition would then presumably activate TGF-β1 which would in turn impact satellite cell function, muscle regeneration, and accumulation of fibrotic, non-contractile tissue (Figure 3). Together these findings suggest that interactions between MALAT1 and miR-34a impact a number of factors such as senescence and fibrosis that are known to drive the aging process in skeletal muscle. Crosstalk between these two non-coding RNAs may therefore represent a potential therapeutic target to alter age-related changes in muscle that contribute to increased disability with aging.

## Data Availability Statement

The original contributions presented in the study are included in the article/supplementary material, further inquiries can be directed to the corresponding author.

## Ethics Statement

The animal study was reviewed and approved by the Augusta University IACUC.

## Author Contributions

LR performed siRNA experiments and analyzed the resulting data. BM and SF analyzed *in vivo* tissue samples. EP and AK performed RNAscope studies. CI, MM-L, and WH assisted with experimental design and data interpretation. MH wrote the final manuscript. All authors contributed to the article and approved the submitted version.

## Conflict of Interest

The authors declare that the research was conducted in the absence of any commercial or financial relationships that could be construed as a potential conflict of interest.

## Publisher’s Note

All claims expressed in this article are solely those of the authors and do not necessarily represent those of their affiliated organizations, or those of the publisher, the editors and the reviewers. Any product that may be evaluated in this article, or claim that may be made by its manufacturer, is not guaranteed or endorsed by the publisher.
